# Teaching, research or balanced? An exploration of the experiences of biomedical scientists working in UK medical schools

**DOI:** 10.1002/2211-5463.13304

**Published:** 2021-10-06

**Authors:** Tracey Collett, Steve Capey, James Edwards, Darrell J. Evans, John C. McLachlan, Helen Watson, David Bristow

**Affiliations:** ^1^ Peninsula Medical School Faculty of Health University of Plymouth UK; ^2^ Swansea University Medical School UK; ^3^ School of Medicine and Public Health The University of Newcastle Callaghan NSW Australia; ^4^ University of Central Lancashire Medical School Preston UK; ^5^ Peninsula Medical School and Peninsula Dental School Faculty of Health University of Plymouth UK

**Keywords:** career, education, medical education, medical school, professional identity

## Abstract

Driven by demand for high standards in university education, efforts have been made in the UK to address the perceived imbalance between teaching and research. However, teaching is still perceived by many as having less credibility and is attributed less importance. The purpose of our research was to explore how distinct types of academic job profiles (‘research’ or ‘education’ focused, or ‘balanced’) impact on biomedical scientists' perceptions of the lecturer role. Specifically, we investigated the experiences of biomedical scientists in ‘post‐1990’ medical schools, which are known for their commitment to excellence in both research and education. We conducted 22 face‐to‐face, semi‐structured interviews with biomedical scientists in five schools. Focusing on experiences of work, the interviews covered: ‘motivations’, ‘role expectations’, ‘teaching’, ‘research’ and ‘career’. The recorded qualitative data were transcribed and then analysed thematically. Our results, offering an insight into the working lives of biomedical scientists in medical education, suggest that in settings with a dual emphasis on education and research, individuals on ‘balanced’ contracts can experience a strong pull between research and teaching. In addition to posing significant challenges with respect to workload management, this can impact profoundly on professional identity. In contrast to the balanced role, ‘research’ or ‘education’ focused roles appear to have clearer requirements, leading to higher employee satisfaction. We conclude that to assist the educational mission of Higher Education, attention should be paid to balanced contracts, to (a) ensure employee support, (b) mitigate against negative perceptions of teaching, and ultimately, (c) guard against staff attrition.

## Graphical abstract

This article, based on interviews with biomedical scientists, explores the experience of working in UK medical schools. As well as providing an insight into the everyday demands of lecturing, it considers three different contract types (‘education’ or ‘research’ focused or ‘balanced’) and asks ‘what are the implications for education and research in settings that stress equal attention to both?’

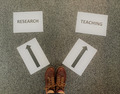

**Abbreviations**
AMS: Academy of Medical Sciences
GMC: General Medical Council
HERA: Higher Education Role Analysis
HESA: Higher Educations Statistics Agency
PBL: problem‐based learning
UKPSF: UK Professional Standards Framework

The past 40 years have seen dramatic changes in Higher Education globally [1, 2, 3]. In the UK the move from ‘fully government funded’ to ‘partly student funded’ education, the increasing role of industry investment in research and a decline in resource provision has led to a new landscape. Through the continued socialisation of graduates and the emphasis on the Research Excellence Framework (REF), the culture of research continues to dominate [4], yet there has been a simultaneous push towards career socialisation, accountability and quality assurance in teaching and learning [5, 6, 7, 8]. The conditions of employment in Higher Education have also shifted noticeably since 2020. COVID‐19 has given rise to new patterns of working (and as a consequence, renewed interest at the institutional level in staff well‐being and care).

In most UK universities, there are three tenured academic contract types or modes of employment. Broadly, individuals are appointed to ‘research’ (‘research fellow’), ‘balanced’ (‘lecturer’, ‘traditional lecturer’) or ‘education’ (‘lecturer education’ or ‘teaching fellow’) contracts. With regard to research or education contracts, the proportion of time spent on research or education, respectively, is high (over 70%), and for those on education contracts, the remaining proportion of time is allocated to the scholarship (study) of teaching and learning. Balanced contracts, on the other hand, assume an approximate 50/50 split between research and teaching [9, 10].

Research exploring the experience of academic employment in Higher Education has tended to focus on job satisfaction, tenure and academic identity (e.g. academic orientation, intrinsic motivating factors and career goals). Studies exploring the identity orientations of individuals in academic careers in science have shown that research tends to be prioritised over teaching. That is, individuals value the ‘independence’, ‘publishing’ and ‘peer recognition’ that an academic career can bring [11, 12]. Moreover, teaching has historically been seen as detrimental to career progression amongst scientists [13] with teaching‐focused academics being less likely to gain promotion or retain their posts [14, 15].

Whilst there has been increasing concern internationally about competition in research *per se* and in particular the ‘publish or perish culture’, especially with respect to age and gender [16], there has been little research into the ‘research only’ career pathway. However, in the United States, a study of public universities by Dugas *et al*. [17] found that amongst those supported to undertake predominantly research, when academic identity and job description aligned, individuals experienced no conflict with regard to their focus, felt relatively unhindered in their progress and had high job satisfaction.

Concerning education‐focused contracts: research suggests that teaching is growing in popularity. In the United States, a survey of life sciences, physics and chemistry doctoral students found that a high proportion were considering teaching‐focused academic careers [17]. In the UK, the Academy of Medical Sciences has reported that there is evidence of academics forming a vibrant community that actively seeks ways of demonstrating excellence in teaching and learning practices [18]. Moreover, research documenting positive experiences of the educator role in medical schools lists identification with and enjoyment of teaching [19] and the experience of working in a setting with individuals from diverse backgrounds as ‘positive gains’ [20].

Despite being the most common form of academic contract in UK Higher Education [10], research into the experience of faculty (staff) working on balanced contracts appears to be limited. However, a number of recent studies have suggested an increasing rise in job dissatisfaction amongst academics because of a perceived tension between teaching and research responsibilities. It is argued that this tension has been exacerbated by increased monetary pressures on universities leading to ‘a change in rhetoric’: universities with a traditional emphasis on education and teaching may now also be compelled to compete in the research arena, or research‐intensive universities may be expected to demonstrate quality in teaching and learning.

For example, with reference to balanced contracts, Dugas *et al*. [17] have argued that researchers who are unable to carve out substantial time to engage in research express the greatest levels of stress. In Finland, Ylijoki [21] describes a divide between faculties, some of whom felt their identities were threatened by institutional changes. In their study of the effect of a new research policy on English Language Teaching lecturers in Vietnam, Anh *et al*. [22] identified a typology of identities including enthusiastic accommodators, pressured supporters, but also ‘losing heart followers’ and ‘disconnected performers’. Similarly, following a study of the experience of academic work undertaken in the United States, Gordon [23] reports that to compensate for a stressful conflict between roles, faculty either worked during their vacation time to meet requirements or prioritised one role over the other (risking job security). In their survey of academic staff working in public (state funded, traditionally teaching focused) universities in Illinois (United States), Dugas *et al*. found that many faculty felt that they were being pulled in too many directions. They argued that something ‘has gotta give’, and they had to do ‘increasingly more with less time’ [24:319].

Like other vocational courses, Medical Schools in the UK are governed by two agencies: the Higher Education sector and a professional regulator [in this case the General Medical Council (GMC)]. Whilst the Higher Education Sector emphasises the importance of both research and education, the profession of medicine regards education as vital to improving the eventual health care of patients [25]. Medical Education, a professional subfield of Medicine, has considerable influence on the design of the medical degree. For example, over the past 40 years, there has been a move towards interdisciplinary, integrated teaching; biomedical (and other) staff are appointed to work within medical school departments; there is an emphasis on social learning processes (advocating small group facilitation and enquiry or problem‐based learning) and the scholarship of teaching and learning is actively encouraged.

The literature suggests that when institutional demands and academic identity align, faculty (academic staff) experience job satisfaction and a sense of personal efficacy and purpose [17]. In contrast, misalignment appears to create dissonance raising significant concerns about staff well‐being and retention and importantly the potential of universities to develop enduring and rigorous programmes of teaching and research for the benefit of the publics that they serve.

The aim of this paper is to contribute to the debate about the experience of academic work, with reference to employment contracts and the balance between teaching and research. Our discussion is based on a qualitative project designed to explore, in‐depth, the perceptions of biomedical scientists working in UK Medical Education.

## Methods

Our research took place between 2011 and 2015. Face‐to‐face interviews were conducted by an experienced qualitative researcher (TC). The object of each interview was ‘a conversation with a purpose’ about participants' perceptions of work. A topic guide was used as an ‘aide memoir’ exploring:

- motivations to work
- expectations of role
- the experience of teaching
- career prospects

The interviews were audio recorded, lasted approximately 1 h and took place at each participant's place of work.

As there was no definitive list of all biomedical scientists working in UK medical schools, we adopted a snowball sampling strategy [26]. Advertising through professional networks, we invited individual biomedical scientists with a managerial role in medical education to participate in the recruitment of further participants. Our final sample was recruited from five ‘post‐1990’ medical schools: schools started in the 1990s following reforms in medical education by the GMC [27] and known for their commitment to education. We emailed invitations to all prospective interviewees identified by the ‘recruiters’ within each school. Thirty individuals (employed at lecturer, senior lecturer or professor level) volunteered to take part, and 22 were interviewed. Of these, 3 participants were employed to undertake ‘mainly research’, 9 were employed on ‘balanced contracts’ (they were expected to develop research and teaching) and 10 were employed on teaching‐focused contracts. Of the research‐focused participants, one was a lecturer and two were senior lecturers; of those on balanced contracts, four were lecturers and five were senior lecturers; and of those on education‐focused contracts, six were lecturers, three were senior lecturers and one was a professor. The gender split is shown in Table 1, and all participants classified themselves using binary terms: 11 were male and 11 were female.

**Table 1:** Participants by career pathway, gender and rank of sample (*n* = 22).

Research focused (80%)						Balanced contract (50/50 split)						Education focused (80%)					
L		SL		P		L		SL		P		L		SL		P	
M	F	M	F	M	F	M	F	M	F	M	F	M	F	M	F	M	F
1	0	1	1	0	0	2	2	2	3	0	0	2	4	2	1	1	0
Total research = 3						Total balanced = 9						Total education = 10					

L, Lecturer (equivalent rank to lecturer A in some Universities); SL, Senior Lecturer (equivalent to Associate Professor or Reader in some Universities); P, Professor.

The interviews were transcribed, anonymised and analysed thematically with the intention of describing the content in‐depth. Three members of the research team (TC, DE and DB) searched six transcripts, and each developed a coding framework. The team then met to negotiate the final framework. Using the qualitative data analysis programme nvivo to organise the data (QSR International Pty Ltd, Doncaster, Vic., Australia), the complete data set was coded to the negotiated frame. As the process was iterative, themes were added as they emerged.

Ethical approval was granted by Plymouth University Ethics Committee and then by each medical school. Adhering to the ethical protocol outlined by the Declaration of Helsinki [28], care was taken to ensure that participants were protected from harm during the project. Participants were informed in detail about the research at least 1 month prior to being interviewed, were assured that confidentiality and anonymity would be respected, notified about the right to withdraw at any point and given contact details. Having been informed, each participant signed a consent form prior to the interview. In reporting the findings, all names have been changed.

## Results

### The research contract

Whilst the interviews were lengthy, the sample of participants on the research only contract was small; however, we have added a short section about the data for purposes of context. All of the participants on research only contracts were happy in their positions. Whilst they said that they enjoyed teaching, they did little of it and were primarily research focused. They felt well supported within their roles and had benefitted from start‐up funding, laboratory space and administrative support. They suggested that it may be less easy to form close collaborations within medical schools as the scientist researchers tended to focus on different areas, respectively; however, a gain was ‘having proximity to clinicians and clinical researchers.’ As Syed states:

… the set‐up here, well is, excellent. There's lots of support for young researchers with the grant writing, … the chief financial officer [is always available] … if I've got any questions the door is always open. There is a dedicated person in the research office, who looks at all the calls coming up and helps you put in applications and kind of gives you a little nudge in the right direction, which is fantastic. And certainly managing budgets and things like that, the finance system here is fantastic. … So all the systems like that seem to be excellent or are excellent. … I got some consumable and start‐up equipment cash, not a huge amount. … I spent the last year writing grants, writing papers, and trying to get the lab going but with nobody actually in the lab except for me, it's difficult to try and juggle the office and lab [but] I have a Ph.D. student starting in October.

### The balanced contract

In the UK, the balanced or ‘traditional lecturer’ role is outlined in the UK Higher Education Standards library of role profiles [9]. As stated above, within this role there is a responsibility for teaching but an institutional requirement to produce scientific outputs that are can be returned in the UK REF. Despite the requirement to teach and research, the interviewees employed to ‘balanced’ contracts saw research as the priority – they were research (not teaching) driven, they associated collectively with established groups of research scientists in the UK and internationally and they were resistant to the idea of identifying primarily as a teacher and scholar. As Peter stated:

… research is my first love and I certainly wouldn't be happy to give that up at all.

The ‘balanced’ science lecturers had arrived at their medical schools following a number of postdoctoral research posts, and working in a medical school was seen as an opportunity to ‘get off the post doc treadmill’, to set up a laboratory and become a principal investigator. Ben said:

This is a proper job, if you like, in the sense that you are responsible for designing the research and you are the Principal Investigator … the responsibility lies with you to build the group and come up with the ideas that will attract funding.

Motivations to work in a medical school included access, through the school or university, to a group of scientists established in the same field of research and new opportunities to undertake translational research. Many had seen working in a medical school as a reasonable route for a research career, and all of the researchers interviewed were aiming for promotion along the research pathway.

Whilst routes to promotion were presumed to be based on producing research outputs, all the research‐focused participants expected to undertake teaching. Teaching duties included responsibility for a scientific theme as well as more generic teaching tasks such as being a module or ‘phase’ lead, leading a special studies component, problem‐based learning (PBL) facilitation, teaching evidence‐based practice and statistics, academic mentoring, public engagement activities and developing educational initiatives for the school.

Importantly, all the participants spoke about the experience of the transition to becoming a lecturer. They emphasised the cognitive load bought about by multitasking and the management of teaching. Educational responsibilities were said to be numerous and diverse. For example, one participant stated that she had 17 discrete teaching‐related projects compared with one research‐related project. Teachers were described as ‘always on’ and required to undertake numerous and varied teaching management‐related tasks on a daily basis such as, organising speakers, rooms, cohorts of students and equipment. In contrast to research, teaching itself and in particular teaching related to PBL type curricula was seen as taking place in ‘stop start’ bursts across the course of the whole academic year.

Whilst participants had taken accredited courses during or prior to their probation, the transition to teaching was generally described as ‘tough’. Particular challenges were as follows: ‘learning how a degree works’; developing a programme, leading a subject for the first time, teaching medical rather than science students, understanding a complex integrated PBL curriculum and learning new approaches to teaching and learning such as self‐direction and facilitation.

The traditional lecturers perceived that in comparison with university science departments, UK medical schools place much higher demands on lecturers to teach. This is something that had not been anticipated. Jack stated:

One of the things that you'll find in most universities where people are working is that they tend to do about a 20/80 split; they do 20% teaching and admin and 80% is research. … My contract is 50/50 research and teaching contract so over the whole year I have to run about 800 hours or teaching related activities.

The experience of working in the balanced role was likened to ‘having two full‐time jobs’. Respondents described working overtime in order to meet the demands of work. In addition, they spoke of ‘being pulled in two directions’ by different managers:

The demands … the quality of research that's expected of us and the teaching load we have is way above what my research peers, competitors, collaborators have to deal with. Someone who is a senior lecturer (in a science department) would not be doing the teaching that I do.

The first few years in the lecturing post were described as particularly difficult. Sarah stated:

It's one of the issues that a lot of us have had with becoming part of the medical school is that when you're that busy setting up the (lab) something's going to give. I've practically half killed myself to keep this research going and keep my fingers in but I think I realised that if I didn't, you're sort of out of sight, out of mind.

A number of the participants talked about the emotional impact of moving into a career that gave what they perceived to be equal weight to teaching and research. For example, Gayle describes her experience of working in a medical school as having two jobs that do not meet. She stated:

The notion of stepping into another career is psychologically very difficult to deal with if it is unexpected, ‘this is not what I trained for, what about my life's work up until this point etc’? I thought I had completely messed up my life, thrown away all of my training.

She continues:

There is also the awful sense of doing something that is kind of unskilled compared to what you were doing in ‘the research state’.

Some of the participants felt detached from the scientific community and that they had been repositioned hierarchically below clinicians. Mariam states:

There is a sense of being in service to medics and some loss with respect to being rooted in a science community and bringing on young scientists. [There is] a disparity between opportunities for clinicians and research scientists at medical schools.

A number of participants mentioned that differential salaries were unfair: Roger states:

Even though the non‐medic maybe significantly more useful in the actual role, the fact is that somebody who is medically qualified often gets a significantly higher salary. That can be a very significant cause of grievance.

A further factor impacting on motivation to teach amongst the traditional lecturers was a perceived lack of reward. Frequent curricula changes or needing to gain approval for a teaching change from multiple stakeholders were said to hamper creativity. Sarah states:

The research is very easy to judge how you're doing because you write grants, you write papers and you see very immediately whether you're doing well. With teaching I could tell you my group love me but short of you getting ethics to go and ask them, you wouldn't know any different. There is no reward for teaching.

Ultimately, for those working in the lecturer role, there was a sense that staying in a medical school could result in ‘career suicide’. As Parel claimed:

I could stay here, it is secure, but it will be the end of a career in science.

### The education contract

The teaching fellow/lecturer role corresponds with the role of ‘teaching fellow’ as outlined in the UK Higher Education Standards (2004) Library of Academic Role Profiles [9]. As stated above, within this role, employees are expected to teach for the majority of the time and undertake educational research or scholarship. Of the teaching fellows/lecturers, three had recently completed their PhDs. The others had moved into medical education following a series of postdoctoral jobs. Motivations for some to take a teaching contract were ‘a sense of disillusionment with a research career’, job security and a better quality of life. All of the respondents were motivated to teach. Teaching offered a different kind of connection with science. A number of participants spoke about deliberately choosing a ‘dual track’ career, about teaching as being exciting and a source of energy as opposed to a ‘distraction from research’. Amongst the interviewees appointed to the education contract, there was a sense of contributing to something new.

Crucially, the teaching‐focused biomedical scientists experienced the practical activity of teaching in the same way as the ‘balanced’ lecturers. For example, a transition, a new emphasis on a multitude of tasks, difference in cognitive load and challenges associated with learning new methods. Additionally, many were critical of the same hierarchical (clinician related) and organisational (reward related) barriers to teaching.

Unlike the majority of the balanced interviewees, however, there was no pull between teaching and research. Instead, educational research or scholarship (occupying on average 20% of the teaching role) was seen as part of the teaching role. A number of participants stated that they aspired to ‘become a medical educator’ or that they were ‘interested in engaging in the pedagogic side of teaching’.

In contrast to the research‐focused biomedical scientists who were negative about educational research, the teachers were more reflexive. Adam states:

One big problem that bench scientists that go into medical education have is that the tendency to move away from quantitative to qualitative measurement of things. XX has been one of the people who has persuaded me that it is a valid method of investigation.

The interview data suggest that individuals better understand educational research or scholarship the longer they have been in a medical school. Rob, a senior lecturer/teaching fellow, states:

… [Research in Medical Education has] a well established series of methodologies. It's been running for long enough to have a track record and to have philosophies within it and there is some outstanding research. Compared to other higher education fields, the pedagogy in medical education is much more advanced. … [Higher Education] is a natural ally.

The findings indicate that (as well as mentorship, and university support) the move into educational research or scholarship may be assisted by attending conferences and other related events. Keith states:

I went to ASME and actually presented what we had done to date on XX. That was interesting because you're always thinking, ‘who's going to be interested? Is it generalised or too focused?’ but it was uplifting in that it was positively received.

In addition, educational research or scholarship may promote a sense of connectedness within an interdisciplinary team as well as align with other teaching activities. Rob states:

I think one big problem for scientists working in a medical school is that they're often on their own. You need a physiologist but you only need one physiologist and therefore they don't have a critical mass. So there's often conflict between research activities and educational activities. If you're doing medical education research then these align. It means that you get groupness, [plus] you get alignment with your day to day activities and you have a higher chance of increasing productivity.

The majority of the teaching‐focused biomedical scientists were satisfied in their posts. They expected to teach and saw teaching as providing a degree of academic freedom. There were numerous examples of innovative scholarship and research in the data. Participants had written books, designed resources, created web‐based teaching programmes, blogs and wikis. They had started to work nationally and internationally with other educators. Moreover, many had started to feed back into their science discipline through the education stream of the relevant association. Claire states:

… I'm on council of the XX I'm constantly fighting to get [our education] profile raised. In the last couple of years they have started to introduce an education symposium into the timetable … I'm sorting out the winter conference. I've booked XXX to come and take it outside of the [names bioscience] context and just challenge their perceptions of education a bit more broadly.

Whilst in general the outlook was positive for those employed to the education role, the senior interviewees complained specifically about career progression. They claimed that routes to promotion had been blocked as there is only a clear ‘track’ for research in their universities. High teaching loads were said to impact on research outputs, and failure to return in the REF was said to lead to the allocation of more teaching. A perceived lack of career equality was seen to cause tension between teachers and researchers, fuelling conceptions of teachers as ‘underdogs’. Paul argues:

You'll never get higher than the senior lecturer unless you've got a strong research record. … There's a huge amount of teaching to be delivered and what tends to happen is you're relying on a smaller group of people doing the bulk of the teaching. If you're never going to give them any career progression, why should they put in that enthusiasm?

In addition, it was argued that within the academic field of medical education, as a scientist (rather than a clinician), it is ‘virtually impossible to progress to Dean’. As Kathy stated:

It's very difficult to go beyond a certain level if you're not medically qualified … the barrier is unbreakable at the moment.

In contrast to the senior respondents, perceptions of promotion were generally positive amongst the newer lecturers. The general perception was that the presence of the (2011) UK Professional Standards teaching profile: (a framework for measuring and progressing in teaching in Higher Education) [7] provides structure and direction to the role. Moreover, in the UK, there is a change in the status of teaching in Higher Education related to changes in funding, increases in student fees and the increasing emphasis on student satisfaction as a measure of university (and medical school) excellence. Teaching in academia was perceived as being ‘rebranded’. Title changes (from ‘teaching fellow' to ‘lecturer’ for example) were seen as indicative of better equality between teachers and researchers. And medical schools were seen as ‘safer’ than science departments as they appeared to invest more heavily in teaching.

## Discussion

This study offers an insight into the various experiences of biomedical science lecturers in UK medical schools, with a particular focus on the challenges encountered in relation to ‘academic role profile’.

Our results suggest that moving into medical education can have a profound impact on individuals with biomedical science backgrounds. Whilst supporting the transition into a lecturer role has been discussed in general and from an instrumental perspective (e.g. with respect to ways of training new lecturers in teaching methods), there has been little discussion about the personal, lived experience of finding oneself ‘apart’ from one's discipline in a new academic and professional cultural setting. Indeed, many of the participants on the ‘balanced’ and the ‘education’ career pathways felt particularly strongly that a study into the working experiences of biomedical scientists in Higher Education was ‘long overdue’ referring, in particular, to the importance of outlining the major personal impact of (a) coming into a medical school from a science department or from a postdoctoral position and (b) progressing into an educational role from doctoral or postdoctoral roles that instil the expectation to that successful academic scientists are funded researchers.

Importantly, our research undertaken within the context of UK medical education highlights the point made by Dugas *et al*. [24] and others, that in settings where dual emphasis is placed on research and teaching, those entrusted to both (those on balanced contracts), particularly at the initial stages of their careers, can experience an overwhelming tension or ‘a sense of a pull’ between tasks. The imperative to understand new pedagogies requires not only an epistemological transition (often difficult to make for biomedical scientists highly schooled in mechanistic thinking and causality and unaccustomed to other forms of reasoning) but also the need to switch between different modalities: the research state, focused and oriented around prolonged periods of time spent on single tasks and the teaching state involving a different kind of cognitive load, including significant multitasking and heavy administration. Crucially, the time spent ‘serving both masters [sic]’ appears to result in a sense of ambiguity with regard to academic identity and less ‘gain’ in terms of output and promotion potential. Notably, the concerns raised by those on the balanced contract about their progress as academics were well founded. In 2020, private correspondence with participants keen to move this work forward revealed that of the original scientists on balanced contracts, seven eventually moved out of the role (and gained promotion) and two remained with no promotion.

The apparent ‘tug of war’ between teaching and research for those on balanced contracts appears to have significant implications for those institutions that expect lecturers to demonstrate impact in both research and teaching. For example, it appears that, when these roles are considered equal, the pull of academic research can override the pull of teaching. This is perhaps not surprising given the culture of academia in general and particularly the focus of doctoral and postdoctoral work. Indeed, the perception of the educator as a poor citizen with little in terms of a metric for success prevailed amongst some participants. For example, one stated ‘those that can't, teach’, and another argued ‘it is the researchers who bring in the money’. Given these circumstances, it seems unlikely that some lecturers on balanced contracts will engage much with the educational mission of the university. As one participant said – ‘teaching is a bit of a chore’.

Yet, a number of participants argued that teaching and research should go hand in hand, echoing the perspective of the Academy of Medical Sciences (AMS) that:

… good teaching inspires students and changes lives, drives the UK's research base leading to a virtuous circle between education and research, one that generates new knowledge and brings health and societal benefits [18: 5]

They argued that a key factor impacting on success in a balanced contract is timing. For example, when research is established, when a lecturer has a team (postdoctoral students, researchers, PhD/MSc students), it is possible to pay more attention to teaching. Other suggestions included organising education in such a way to minimise the impact (e.g. ‘lumping’ teaching together), incorporating ideas about the value of teaching into secondary, further and Higher Education and post doc training, highlighting the value of transferable skills courses for doctoral students and developing exemplar profiles for medical education of individuals who successfully maintain a balanced career. These suggestions are hopeful: in settings that favour research and education equally, rather than abandon the balanced role, it may be possible to adjust it.

In contrast perhaps to the balanced role, a striking finding from our study is that the educator role appears to be particularly well supported in institutions that emphasise both education and research. For those interviewed, the education pathway was a rewarding and creative career option. Congruent with the findings of Nikendei *et al*. and Hu *et al*. [19, 20], our results suggest that participants on the educator pathway impacted on education through a broad range of projects. These included the following: writing and updating text books, setting up best practice networks, creating online resources, working on issues related to mentoring, pastoral care and learning with patients and in the community. Participants were developing learning in small group settings, initiating outreach activities in widening access to medicine and projects associated with the public understanding of science. They were working on theoretical understandings of education, for example, dialogic processes in education or social learning theory. Importantly (and resonating with the idea of the virtuous circle cited in the AMS report above), they were also involved in reviving or creating new links between teaching and research particularly with respect to setting up active teaching networks within biomedical societies. Such activities were (or had the potential to be) feeding into both the practice and research agendas of the academic field of Higher Education [2].

One question arising from this research is whether medical schools offer better opportunities for educators than other university departments. Of the original participants on educator contracts, those at lecturer level were, over time, promoted to senior lecturer positions within medical education, suggesting a degree of career mobility. However, it may be the case that in terms of promotion, the educator pathway is more risky. Are, for example, opportunities for educators more likely to fluctuate as, over time, demands regarding the role of the lecturer change? And in Medical Schools are ‘lecturer requirements’ subject to a broader tug of war between the sectors of Higher Education and medicine? Certainly, the comment made by Kathy above appears to be borne out by HESA data [10]. This suggests that, in general, those on balanced and research contracts are more likely to attain higher academic positions.

Finally in terms of developing their own role profiles and identities as educators, the existence of the UK Professional Standards for teaching (UKPSF) was seen as useful, providing frameworks for portfolios of practice and arguments for reward. Other motivating factors were support for scholarship both from within the field of medical education and Higher Education and more broadly narratives foregrounding ‘public science’, ‘interdisciplinarity’, ‘transferability’, ‘scholarship’, ‘integration’ and ‘collaboration’.

### Study limitations, further research and conclusion

With respect to the generalisability of our results, the research sample for this study was ‘individuals from a selection of UK medical schools’ and we cannot claim that our findings are representative of all medical schools or scientists (particularly those outside of the UK). Experiences may be different for different individuals, and experiences may vary according to the faculty structure of the school, its teaching philosophy and the university within which it resides. In order to minimise bias, care was taken to ensure that the interviewer was unknown to the respondents in the study and respondents were ensured of anonymity and confidentiality; however, respondents may have withheld comments and thoughts due to being nominated by ‘someone they knew’. Further, whilst care was taken to check against interviewer bias, the interviewer (herself a social science lecturer/teaching fellow) may have influenced the participants (and analysis) by prompting (or searching) for particular answers.

This study is not indicative of all medical schools, and we do not claim to generalise to Higher Education more broadly or assume that the situation is the same for all faculty on research, balanced or education contracts. Our paper is based on the premise that the experience of academic work should not be treated as a single entity. Individual circumstances are influenced by structural conditions (e.g. cultural norms and governmental policies). Conditions are mitigated by contract type/role profile and by institutional ethos and orientation. Moreover, they are influenced by departmental policies and values and subject to individual variation (e.g. tenure and experience). However, it is possible to ‘hold these variables still’ and notice properties of work that resonate with groups of academics at particular points in time. Our main point is in agreement with Dugas *et al*. that in general, departments that prioritise teaching and research equally may need to pay close attention to the impact on staff. With respect to biomedical scientists working in medical education, these individuals make up a significant proportion of the work force, yet have received very little attention. Neglecting the experiences of staff has implications not only for well‐being and retention but also for the quality of teaching and research.

Future research might seek to follow up these findings through quantitative questionnaire surveys that test the key themes in a large population and cross‐reference the findings with ‘institution type’, ‘role profile’ and ‘individual characteristics’. Equally important, a series of localised qualitative studies might usefully examine in‐depth similar and different cohorts of biomedical scientists to explore whether the themes are replicated and the nuances that different settings can engender.

## Conflict of interest

The authors declare no conflict of interest.

## Author contributions

TC, DB and DJE conceived and designed the original project; TC collected the data; TC, DB and DJE worked on the initial analysis of the data; JCM contributed at several stages of the initial project including recruitment and ethics; TC, JE and HW conceived of this specific paper and worked together on early drafts. TC wrote the final paper, all authors commented and helped to shape the final draft.

## Data Availability

The data are available on request due to privacy/ethical restrictions. Please contact the corresponding author.
